# Tomato Brown Rugose Fruit Virus Evades Tm‐2^2^‐Mediated Resistance by Avoiding the Induction of Tm‐2^2^ Self‐Association

**DOI:** 10.1111/mpp.70243

**Published:** 2026-03-19

**Authors:** Hua‐Yu Ma, Yi‐Jie Liu, Xiao‐Xue Huan, Shu‐Yu Liu, Xiu‐Qi Mu, Li‐Meng Zhang, Zhi‐Long Bao, Yan‐Ping Tian, Zhi‐Yong Yan, Xiang‐Dong Li

**Affiliations:** ^1^ Department of Plant Pathology, College of Plant Protection Shandong Agricultural University Tai'an Shandong China; ^2^ College of Horticulture Science and Engineering Shandong Agricultural University Tai'an Shandong China; ^3^ Shandong Key Laboratory for Green Prevention and Control of Agricultural Pests, Institute of Plant Protection Shandong Academy of Agricultural Sciences Ji'nan Shandong China

**Keywords:** evasion mechanism, hypersensitive response, movement protein, Tm‐2^2^ self‐association, ToBRFV

## Abstract

Tomato brown rugose fruit virus (ToBRFV) overcomes Tm‐2^2^‐mediated resistance, posing a serious threat to global tomato production. We previously identified that key residues in the movement protein (MP)‐H67, N125, K129, A134, I147 and I168 are essential for ToBRFV evasion of Tm‐2^2^ resistance. However, the underlying mechanisms remain unclear. In *Tm‐2* ^*2*^‐transgenic *Nicotiana benthamiana* plants at later infection stages, MP mutations H67C, N125A, K129Q and A134N abolished ToBRFV‐GFP cell‐to‐cell and systemic movement, whereas I168N abolished ToBRFV‐GFP systemic but not cell‐to‐cell movement, and I147M partially impaired both. Correspondingly, the MP^H67C^, MP^N125A^, MP^K129Q^ and MP^A134N^ mutants elicited strong *Tm‐2* ^*2*^‐mediated hypersensitive response (HR), MP^I168N^ elicited mild HR and MP^I147M^ elicited none. Confocal microscopy revealed I147M reduced MP localisation at plasmodesmata in *Tm‐2* ^*2*^‐transgenic *N. benthamiana* leaves. The interaction with Tm‐2^2^ was weak for wild‐type MP and I147M (slightly enhanced), but moderately or strongly enhanced for I168N and the other four mutants. Wild‐type MP and MP^I147M^ failed to induce Tm‐2^2^ self‐association, MP^I168N^ only weakly induced it, whereas the other four markedly induced it. Collectively, these findings demonstrate that ToBRFV evades Tm‐2^2^‐mediated resistance by attenuating MP–Tm‐2^2^ interaction strength, thereby avoiding Tm‐2^2^ self‐association and downstream immune activation.

## Graphical abstract

Tomato brown rugose fruit virus evades Tm‐2^2^‐mediated resistance by reducing affinity between movement protein and Tm‐2^2^, thus failing to trigger Tm‐2^2^ self‐association and activation of immune signalling, while maintaining MP function to support viral systemic infection.

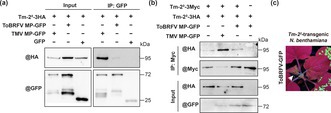

## 1 Introduction

Plants have evolved nucleotide‐binding leucine‐rich repeat receptor (NLR)‐based immunity against pathogens (Jones and Dangl 2006; Zhou and Zhang 2020). Intracellular NLRs‐CNLs (coiled‐coil) or TNLs (TIR) detect effectors to trigger effector‐triggered immunity (ETI) (Jones et al. 2016; Li et al. 2015; Saur et al. 2021).

Tomato Tm‐2^2^, a CNL resistance protein, recognises movement proteins (MPs) of tobacco mosaic virus (TMV), tomato mosaic virus (ToMV) and tomato mottle mosaic virus (ToMMV) to confer resistance (Lanfermeijer et al. 2003; Tettey et al. 2023). Tm‐2^2^‐mediated resistance depends on its dosage, host factors (RuBisCO, MIP1) and temperature (Du et al. 2013; Tettey et al. 2023; Zhang et al. 2013; Zhao et al. 2013). Tm‐2^2^ interacts with TMV MP at the plasma membrane (PM), where MP recognition triggers NB‐ARC‐regulated self‐association that is essential for the hypersensitive response (HR) (Chen et al. 2017; Wang et al. 2020).

*Tm‐2* ^*2*^ is extensively used in tomato breeding against tobamoviruses, but resistance‐breaking isolates and emerging viruses overcome it. For instance, ToMV MP mutant (S238R/K244E, D240Y) evades Tm‐2^2^‐mediated HR and resistance (Kuroiwa et al. 2022; Weber et al. 1993; Zhang et al. 2013). Recently emerged tomato brown rugose fruit virus (ToBRFV) also infects *Tm‐2* ^*2*^‐harbouring tomatoes, threatening global tomato production (Luria et al. 2017; Yan, Zhao, et al. 2021). ToBRFV MP has been identified as the key virulence determinant for ToBRFV to infect *Tm‐2* ^*2*^‐carrying tomato plants (Hagit and Ziv 2021; Yan, Ma, et al. 2021). We previously demonstrated that six central MP residues (H67, N125, K129, A134, I147 and I168) are critical for evading Tm‐2^2^‐mediated resistance (Yan, Ma, et al. 2021). ToBRFV MP also hijacks the RabE1a/SEC10 secretory pathway and AP2β endocytic pathway for movement (Ma et al. 2025). TMV MP C68H (corresponding to ToBRFV MP H67) reduces plasmodesmata (PD) localisation and cell‐to‐cell movement (Hak et al. 2023). However, ToBRFV evasion mechanisms remain unclear; elucidating them will be helpful to design effective control strategies.

In this study, we demonstrate that ToBRFV evades Tm‐2^2^‐mediated resistance by weakening the MP–Tm‐2^2^ interaction, thereby avoiding Tm‐2^2^ self‐association and immune activation.

## 2 Results

### 2.1 H67, N125, K129, A134, I147 and I168 of MP Are Essential for ToBRFV‐GFP Systemic Spread in *Tm‐2^2^*‐Transgenic *Nicotiana benthamiana*

**FIGURE 1:**
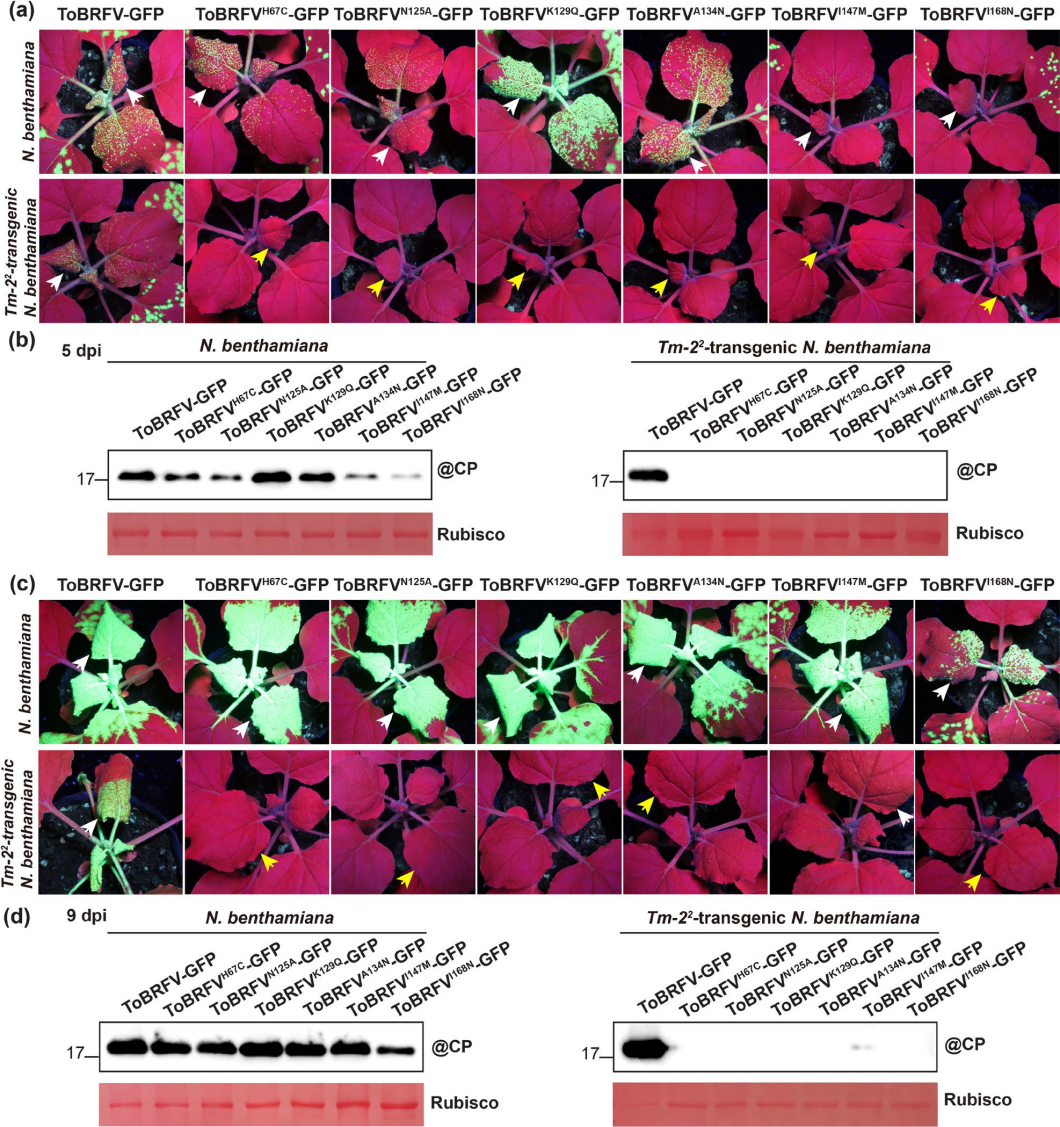
Effect of point mutations on ToBRFV‐GFP systemic movement in wild‐type and *Tm‐2* ^*2*^‐transgenic *Nicotiana benthamiana* plants. (a) GFP fluorescence in systemic leaves of wild‐type (upper) and *Tm‐2* ^*2*^‐transgenic (lower) *N. benthamiana* plants at 5 days post‐inoculation (dpi). White and yellow arrowheads indicate detectable and undetectable fluorescence, respectively. Images were captured under UV illumination. (b) Western blotting analysis of coat protein (CP) accumulation in systemic leaves at 5 dpi of wild‐type (left panel) and *Tm‐2* ^*2*^‐transgenic (right panel) *N. benthamiana* plants inoculated with ToBRFV‐GFP or virus mutants. Anti‐ToBRFV CP antibody was used for detection. Ponceau S‐staining of RuBisCO served as loading control. (c) GFP fluorescence in systemic leaves at 9 dpi. (d) Western blotting analysis of CP accumulation in systemic leaves at 9 dpi. Plants were inoculated at 3–4 weeks old. For each treatment, three plants were used per treatment, and the experiment was independently repeated three times.

Our previous study showed that ToBRFV^H67C^, ToBRFV^N125A^, ToBRFV^K129Q^, ToBRFV^A134N^, ToBRFV^I147M^ and ToBRFV^I168N^ systemically infect wild‐type *N. benthamiana* but not *Tm‐2* ^*2*^‐transgenic *N. benthamiana* at 5 days post‐infiltration (dpi). To dissect residues' roles in movement, we individually introduced these mutations into a ToBRFV‐GFP infectious clone's MP, generating ToBRFV^H67C^‐GFP, ToBRFV^N125A^‐GFP, ToBRFV^K129Q^‐GFP, ToBRFV^A134N^‐GFP, ToBRFV^I147M^‐GFP and ToBRFV^I168N^‐GFP. We agroinfiltrated wild‐type and *Tm‐2* ^*2*^‐transgenic *N. benthamiana* with ToBRFV‐GFP or mutants, assessing systemic infection and coat protein (CP) accumulation at 5 and 9 dpi, respectively (Figure 1).

At 5 dpi, ToBRFV‐GFP showed green fluorescence in wild‐type *N. benthamiana* leaves, whereas ToBRFV^K129Q^‐GFP and ToBRFV^A134N^‐GFP produced markedly stronger fluorescence (Figure 1a, upper panel). Conversely, ToBRFV^H67C^‐GFP, ToBRFV^N125A^‐GFP, ToBRFV^I147M^‐GFP and ToBRFV^I168N^‐GFP produced weaker fluorescence (Figure 1a, upper panel). Western blots confirmed higher CP for ToBRFV^K129Q^‐GFP and ToBRFV^A134N^‐GFP and lower for others versus wild‐type (Figure 1b, left panel). These data indicate that K129Q and A134N enhance, while H67C, N125A, I147M and I168N delay, systemic movement in wild‐type *N. benthamiana* plants.

At 5 dpi, in *Tm‐2* ^*2*^‐transgenic *N. benthamiana* plants, no mutants showed fluorescence whereas ToBRFV‐GFP did (Figure 1a, lower panel). Western blots detected CP only in ToBRFV‐GFP‐infected plants (Figure 1b, right panel). These results indicate that all six mutations abolished systemic movement in *Tm‐2* ^*2*^‐transgenic *N. benthamiana* plants, confirming our previous findings.

At 9 dpi in wild‐type *N. benthamiana* plants, ToBRFV‐GFP and all viral mutants except ToBRFV^I168N^‐GFP showed strong fluorescence (Figure 1c, upper panel). Western blots confirmed similar CP accumulation levels for all except lower ToBRFV^I168N^‐GFP (Figure 1d, left panel).

At 9 dpi, strong green fluorescence was observed in *Tm‐2* ^*2*^‐transgenic plants infected with ToBRFV‐GFP, whereas plants inoculated with ToBRFV^I147M^‐GFP produced only weak fluorescence. No detectable fluorescence was observed for any of the other viral mutants (Figure 1c, lower panel). Western blots showed reduced CP accumulation in ToBRFV^I147M^‐GFP‐infected plants relative to ToBRFV‐GFP, while CP was undetectable in plants inoculated with the remaining mutants (Figure 1d, right panel). Together, these results indicate that Tm‐2^2^‐mediated resistance restricts the systemic movement of ToBRFV mutants, although the I147M substitution in MP partially compromises but does not completely abolish viral movement in *Tm‐2* ^*2*^‐transgenic *N. benthamiana* plants.

### 2.2 H67, N125, K129, A134, I147 and I168 of MP Are Critical for ToBRFV Cell‐To‐Cell Movement in *Tm‐2^2^*‐Transgenic *N. benthamiana* Plants

**FIGURE 2:**
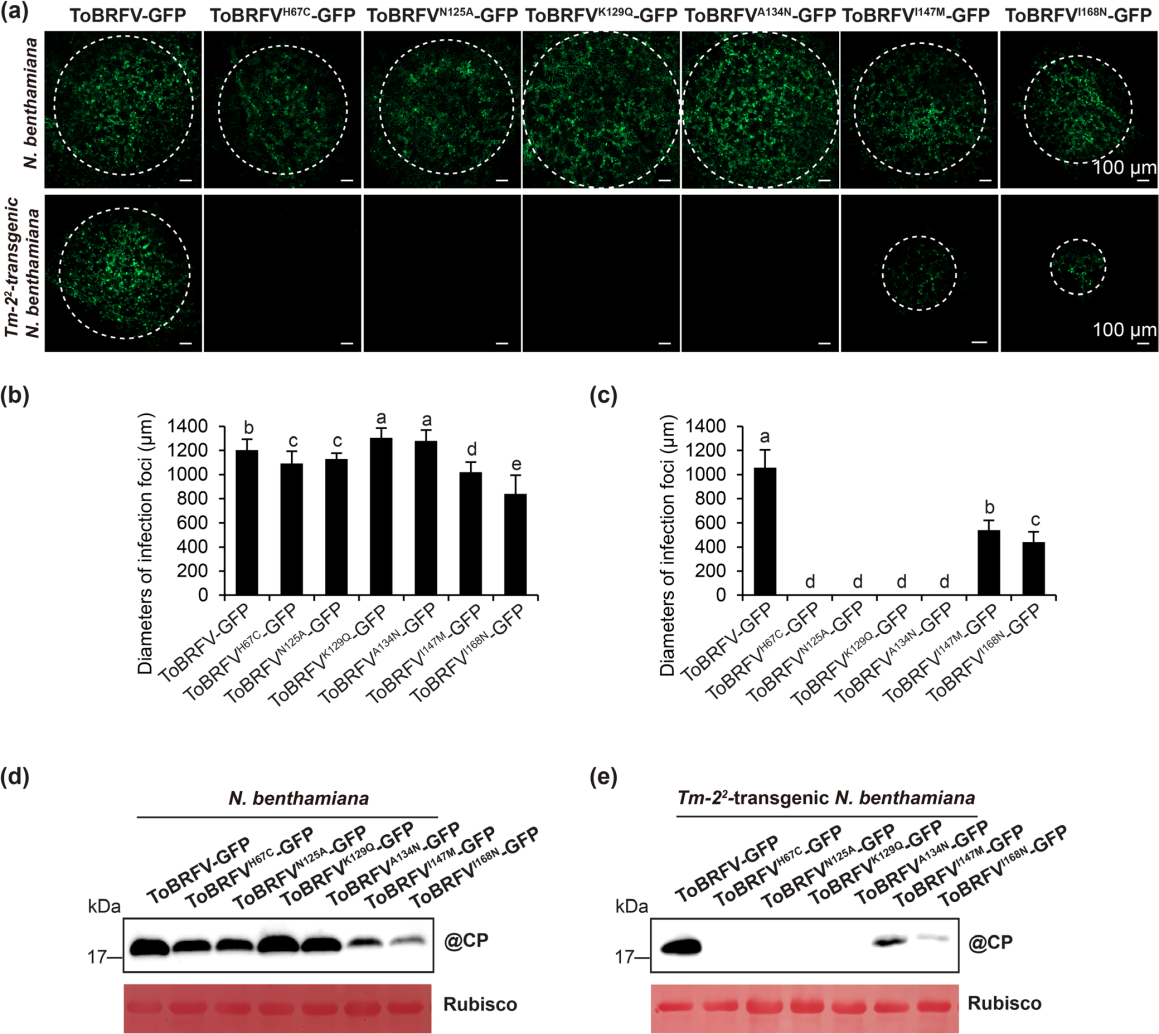
MP mutations impair ToBRFV‐GFP cell‐to‐cell movement in wild‐type and *Tm‐2* ^*2*^‐transgenic *Nicotiana benthamiana* plants. (a) GFP fluorescence in inoculated leaves of wild‐type and *Tm‐2* ^*2*^‐transgenic *N. benthamiana* plants at 72 h post‐inoculation (hpi). Scale bars, 100 μm. (b, c) Quantification of infection foci size in (a), upper and lower panel. Error bars, mean ± SD of 15 images. Different letters denote significant differences (Duncan's multiple range test, *p* < 0.05). (d, e) Western blots of coat protein (CP) accumulation in wild‐type (d) and *Tm*‐*2* ^*2*^‐transgenic (e) plants at 72 hpi. Anti‐ToBRFV CP antibody was used for detection. Ponceau S staining of RuBisCO served as a loading control. Plants were inoculated at 3–4 weeks old. For each treatment, three plants were used per treatment, and the experiment was independently repeated three times.

To investigate the role of MP residues H67, N125, K129, A134, I147 and I168 in ToBRFV cell‐to‐cell movement, wild‐type and *Tm‐2* ^*2*^‐transgenic *N. benthamiana* plants were rub‐inoculated with leaf extracts containing ToBRFV‐GFP or its mutants. At 72 h post‐inoculation (hpi), ToBRFV^K129Q^‐GFP and ToBRFV^A134N^‐GFP produced significantly larger infection foci in wild‐type *N. benthamiana* plants compared to ToBRFV‐GFP; foci of ToBRFV^H67C^‐GFP and ToBRFV^N125A^‐GFP were smaller, those of ToBRFV^I147M^‐GFP even smaller, and those of ToBRFV^I168N^‐GFP produced the smallest foci (Figure 2a upper panel, Figure 2b). Western blotting analysis confirmed these differences in CP accumulation levels across the mutants (Figure 2d). These results demonstrate that K129Q and A134N enhance, while H67C, N125A, I147M and I168N delay, ToBRFV cell‐to‐cell movement in wild‐type *N. benthamiana*.

At 72 hpi, no GFP fluorescence was observed in leaves of *Tm‐2* ^*2*^‐transgenic *N. benthamiana* plants inoculated with ToBRFV^H67C^‐GFP, ToBRFV^N125A^‐GFP, ToBRFV^K129Q^‐GFP or ToBRFV^A134N^‐GFP (Figure 2a lower panel). Infection foci of ToBRFV^I147M^‐GFP were significantly larger than those of ToBRFV^I168N^‐GFP, but the infection foci of both mutants were markedly smaller than those of ToBRFV‐GFP (Figure 2c). Western blotting results were consistent with these observations (Figure 2e). These results suggest that Tm‐2^2^ confers resistance to ToBRFV mutants by abolishing or interfering with their cell‐to‐cell movement, but ToBRFV evades Tm‐2^2^‐mediated resistance to viral cell‐to‐cell movement.

At 72 hpi, the diameter of ToBRFV infection foci in wild‐type *N. benthamiana* was significantly larger than that in *Tm‐2* ^*2*^‐transgenic *N. benthamiana* (Figure 2a–c). These results indicate that Tm‐2^2^ confers partial resistance to ToBRFV by delaying viral cell‐to‐cell movement.

### 2.3 H67, N125, K129, A134 and I168 of ToBRFV MP Are Critical for Avoiding the Activation of Tm‐2^2^‐Mediated HR

Observing abolished or delayed movement of ToBRFV mutants in *Tm‐2* ^*2*^‐transgenic plants, we hypothesised that these MP mutants might be recognised by Tm‐2^2^, thereby activating Tm‐2^2^‐mediated resistance. To test this, wild‐type MP‐GFP and six mutants were expressed via *Agrobacterium* in leaves of *Tm‐2* ^*2*^‐transgenic *N. benthamiana* plants to assess HR.

**FIGURE 3:**
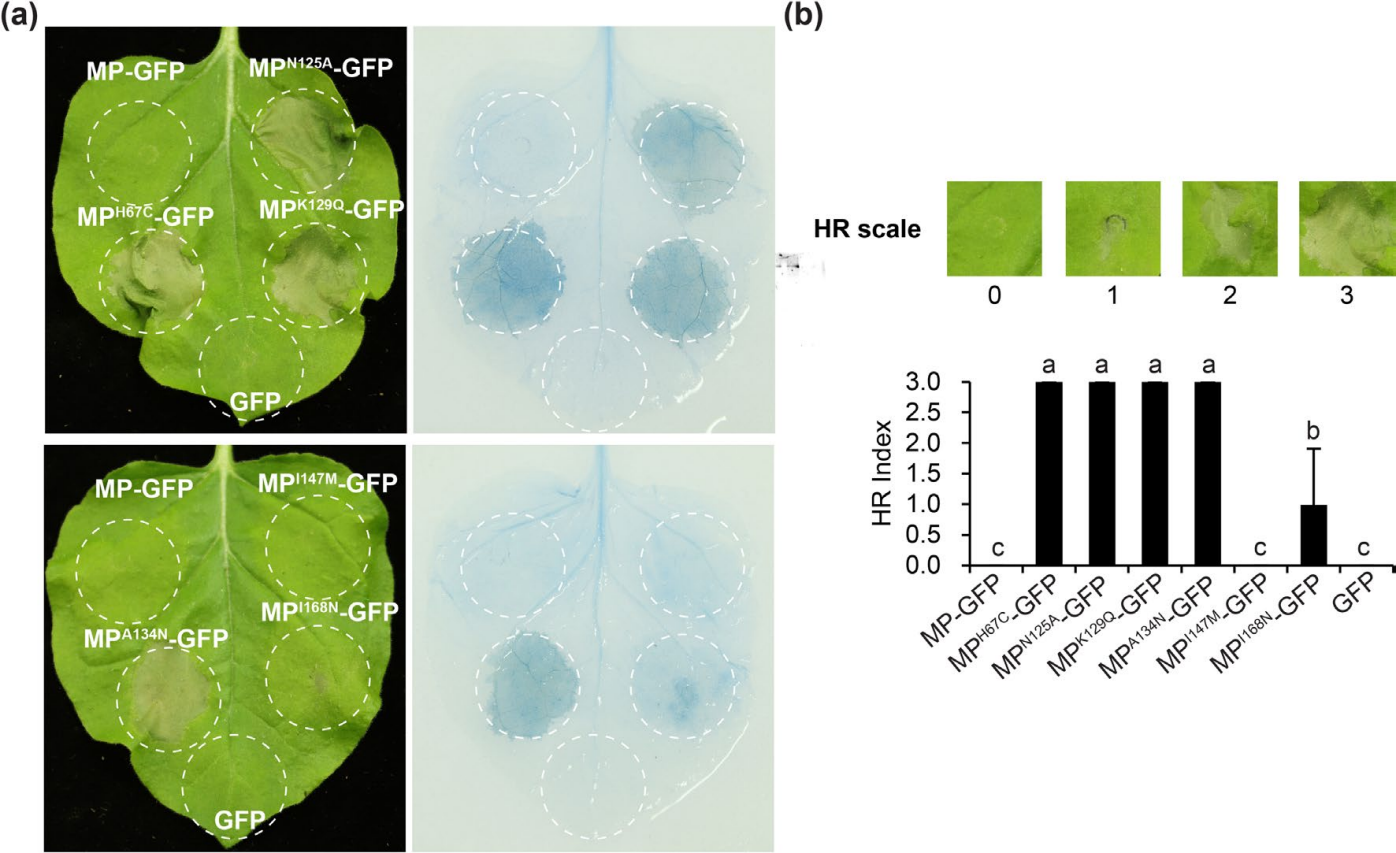
Effect of ToBRFV MP mutations on Tm‐2^2^‐mediated hypersensitive response (HR)‐associated cell death. (a) HR‐associated cell death induced by MP‐GFP, MP^H67C^‐GFP, MP^N125A^‐GFP, MP^K129Q^‐GFP, MP^A134N^‐GFP, MP^I147M^‐GFP and MP^I168N^‐GFP in *Tm‐2* ^*2*^‐transgenic *Nicotiana benthamiana* leaves at 2 days post‐inoculation (dpi). GFP expression was used as a negative control. Infiltrated leaves were stained with trypan blue at 2 dpi to evaluate cell death. (b) HR intensity was scored on a scale from 0 (no cell death) to 3 (severe cell death) at 2 dpi. Data are presented as the mean ± SD from 15 biological replicates. Different letters indicate statistically significant differences, as determined by Duncan's multiple range test (*p* < 0.05). Plants were agroinfiltrated at 3–4 weeks old. For each treatment, three plants were used per treatment, and the experiment was independently repeated three times.

At 2 dpi, leaf patches expressing MP^H67C^‐GFP, MP^N125A^‐GFP, MP^K129Q^‐GFP or MP^A134N^‐GFP showed strong HR cell death, whereas MP^I168N^‐GFP displayed lower intensity (Figure 3a,b). Patches expressing MP^I147M^‐GFP or wild‐type MP‐GFP elicited no visible HR (Figure 3a,b). Trypan blue staining confirmed the extent of cell death (Figure 3a). These results indicate that residues H67, N125, K129, A134 and I168 are critical for evading Tm‐2^2^‐mediated HR.

### 2.4 I147 is Involved in ToBRFV MP Localisation to PD in *Tm‐2^2^*‐Transgenic *N. benthamiana* Plants

**FIGURE 4:**
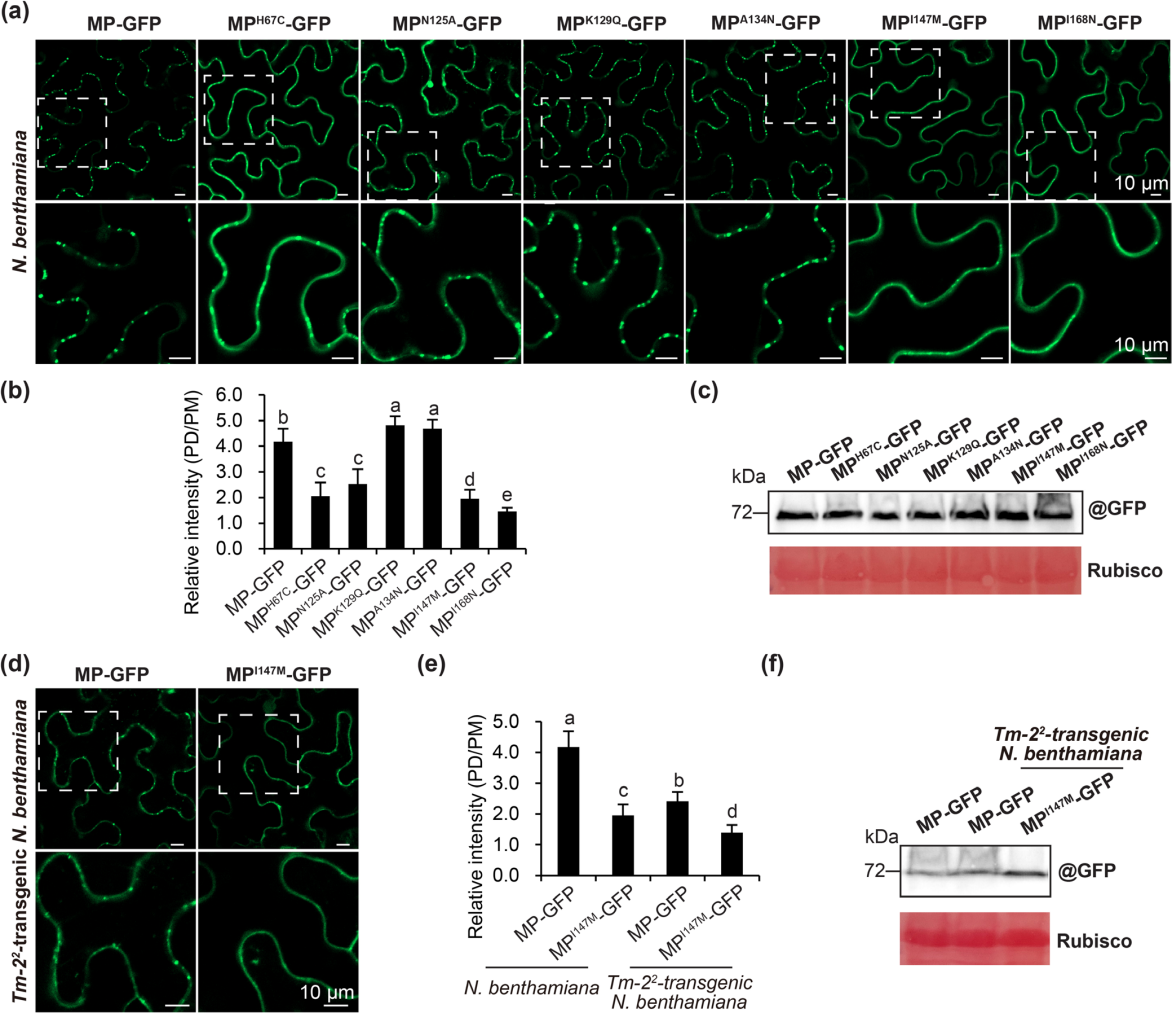
Effect of ToBRFV MP mutations on subcellular localisation of MP‐GFP in wild‐type and *Tm‐2* ^*2*^‐transgenic *Nicotiana benthamiana* plants. (a) Confocal images showing subcellular localisation of MP‐GFP, MP^H67C^‐GFP, MP^N125A^‐GFP, MP^K129Q^‐GFP, MP^A134N^‐GFP, MP^I147M^‐GFP and MP^I168N^‐GFP in the leaves of wild‐type *N. benthamiana* plants at 48 h post‐infiltration (hpi). (b) Relative GFP fluorescence intensity at plasmodesmata (PD) and plasma membrane (PM) in leaves of wild‐type *N. benthamiana* plants. The bar graphs represent the mean ± SD of 80 PD spots from eight images. Different letters indicate significant differences (Duncan's test, *p* < 0.05). (c) Western blotting analysis of accumulation of MP‐GFP and its mutants in inoculated leaves of wild‐type *N. benthamiana* plants at 2 days post‐inoculation (dpi). Ponceau S staining of RuBisCO served as a loading control. (d) Confocal images showing subcellular localisation of MP‐GFP and MP^I147M^‐GFP in the leaves of *Tm‐2* ^*2*^‐transgenic *N. benthamiana* plants at 48 hpi. (e) Relative GFP fluorescence intensity at PD and PM in the leaves of *Tm‐2* ^*2*^‐transgenic *N. benthamiana* plants. Bar graphs represent the mean ± SD of 80 PD spots from eight images. (f) Western blotting analysis of MP‐GFP and MP^I147M^‐GFP accumulation in leaves of *Tm‐2* ^*2*^‐transgenic *N. benthamiana* plants and MP‐GFP expression levels in the leaves of *Tm‐2* ^*2*^‐transgenic *N. benthamiana* plants at 2 dpi. Ponceau S staining of RuBisCO served as a loading control. Plants were agroinfiltrated at 3–4 weeks old. For each treatment, three plants were used per treatment, and the experiment was independently repeated three times.

To investigate whether substitution at residues H67, N125, K129, A134, I147 and I168 influence PD localisation of MP, mutations H67C, N125A, K129Q, A134N, I147M and I168N were introduced into pCamToBRFV MP‐GFP. These constructs and wild‐type MP‐GFP were transiently expressed via agroinfiltration in the leaves of wild‐type *N. benthamiana* plants. At 48 hpi, confocal microscopy revealed higher relative PD/PM fluorescence intensities of MP^K129Q^‐GFP and MP^A134N^‐GFP than MP‐GFP, whereas intensities for MP^H67C^‐GFP, MP^N125A^‐GFP, MP^I147M^‐GFP or MP^I168N^‐GFP were lower (Figure 4a,b). Western blotting confirmed comparable expression levels (Figure 4c). These results indicate that K129Q and A134N substitutions promote, whereas H67C, N125A, I147M and I168N restrict, ToBRFV MP targeting to PD in wild‐type *N. benthamiana* plants.

To assess whether I147M mutation further restricts the PD localisation of ToBRFV MP in the presence of *Tm‐2* ^*2*^, wild‐type MP‐GFP and MP^I147M^‐GFP were expressed in *Tm‐2* ^*2*^‐transgenic *N. benthamiana* leaves. Confocal analysis showed lower PD/PM fluorescence intensities for both MP‐GFP and MP^I147M^‐GFP in *Tm‐2* ^*2*^‐transgenic versus wild‐type *N. benthamiana* leaves (Figure 4d,e). Western blots indicated equivalent expression (Figure 4c,f). These data demonstrate that Tm‐2^2^ restricts MP^I147M^‐GFP PD targeting but insufficiently to block cell‐to‐cell and systemic movement in *Tm‐2* ^*2*^‐transgenic *N. benthamiana*.

### 2.5 ToBRFV MP Residues H67, N125, K129, A134 and I168 Play Critical Roles in Weakening ToBRFV MP–Tm‐2^2^ Interaction, Enabling Resistance Evasion

**FIGURE 5:**
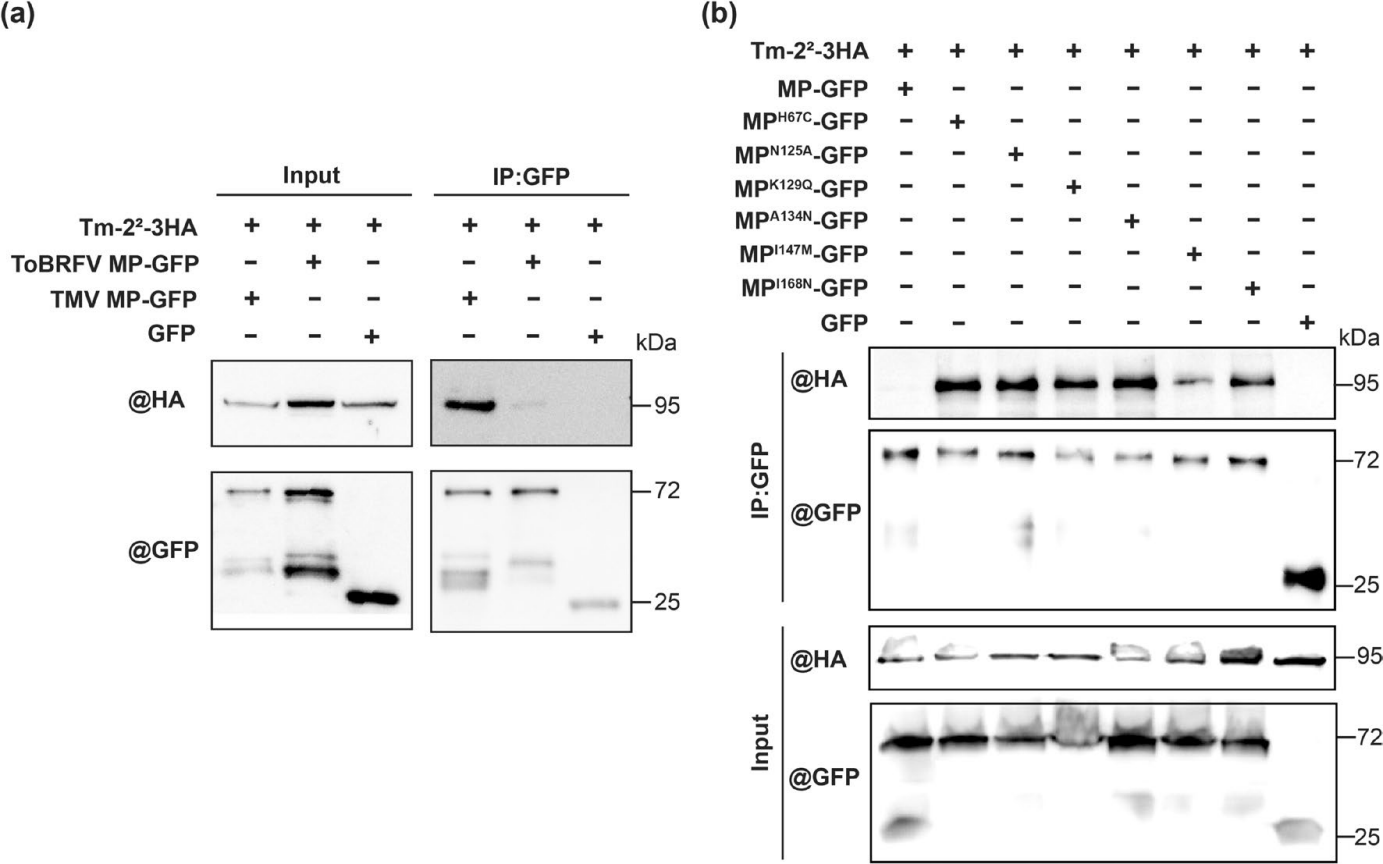
Effects of ToBRFV MP mutations on interaction with Tm‐2^2^. (a) Co‐immunoprecipitation showing Tm‐2^2^‐3HA interaction with tobacco mosaic virus (TMV) MP‐GFP (positive control), tomato brown rugose fruit virus (ToBRFV) MP or GFP (negative control) in *Nicotiana benthamiana* leaves at 24–26 h post‐inoculation (hpi). (b) Co‐immunoprecipitation showing Tm‐2^2^‐3HA interactions with wild‐type or ToBRFV MP mutants at 24–26 hpi. Co‐expression of Tm‐2^2^‐3HA and GFP served as a negative control. Total proteins were immunoprecipitated (IP) with anti‐GFP beads. Both input proteins and IP proteins were immunoblotted (IB) with anti‐GFP or anti‐HA antibodies. Plants were agroinfiltrated at 3–4 weeks old. For each treatment, three plants were used per treatment, and the experiment was independently repeated three times.

To investigate ToBRFV MP evasion of Tm‐2^2^‐mediated HR, we examined ToBRFV MP–Tm‐2^2^ interaction using co‐immunoprecipitation (Co‐IP). Tm‐2^2^‐3HA was co‐expressed with ToBRFV MP‐GFP, TMV MP‐GFP or GFP in *N. benthamiana* leaves, harvested at 24 hpi when slight cell death appeared. Results showed that TMV MP‐GFP strongly co‐immunoprecipitated with Tm‐2^2^‐3HA, whereas ToBRFV MP‐GFP showed weak interaction (Figure 5a). These results indicate that weak ToBRFV MP‐Tm‐2^2^ interaction fails to activate Tm‐2^2^‐mediated HR.

We next used Co‐IP to assess whether mutations H67C, N125A, K129Q, A134N, I147M and I168N in MP enhance the interaction with Tm‐2^2^. All six MP‐GFP mutants showed stronger association with Tm‐2^2^‐3HA than wild‐type MP‐GFP (Figure 5b). Notably, MP^H67C^‐GFP, MP^N125A^‐GFP, MP^K129Q^‐GFP and MP^A134N^‐GFP exhibited markedly stronger binding, MP^I168N^‐GFP showed moderate enhancement, and MP^I147M^‐GFP showed the weakest (Figure 5b). These findings indicate that residues H67, N125, K129, A134 and I168 are essential for weakening the ToBRFV MP–Tm‐2^2^ interaction to evade HR activation. I147 additionally contributes to attenuation of the interaction and relieves Tm‐2^2^ restriction of MP PD localisation.

### 2.6 Residues H67, N125, K129, A134 and I168 of ToBRFV MP Are Critical for Avoiding the Induction of Tm‐2^2^ Self‐Association

**FIGURE 6:**
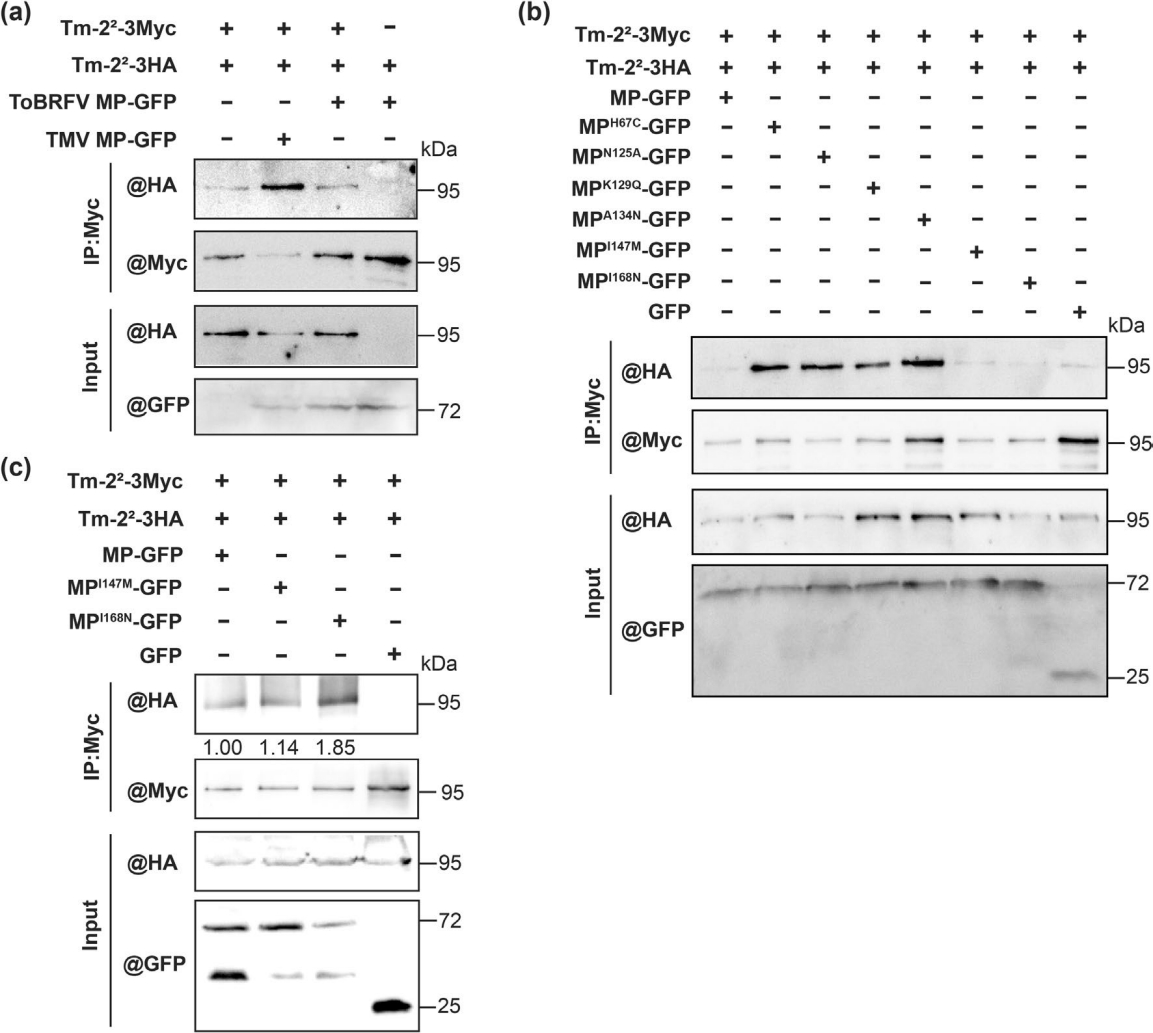
Effects of H67C, N125A, K129Q, A134N, I147M and I168N substitutions in ToBRFV MP on Tm‐2^2^ self‐association. (a) Co‐immunoprecipitation (Co‐IP) analysis of Tm‐2^2^ self‐association in the presence of tobacca mosaic virus (TMV) MP‐GFP, tomato brown rugose fruit virus (ToBRFV) MP‐GFP or empty vector. *Nicotiana benthamiana* leaves co‐expressing Tm‐2^2^‐3HA and Tm‐2^2^‐3Myc together with TMV MP‐GFP, ToBRFV MP‐GFP or empty vector were harvested at 24–26 h post‐inoculation (hpi). Co‐expression Tm‐2^2^‐3Myc with ToBRFV MP‐GFP served as a negative control. (b) Co‐IP analysis of Tm‐2^2^ self‐association in the presence of wild‐type or ToBRFV MP mutants. *N. benthamiana* leaves co‐expressing Tm‐2^2^‐3HA and Tm‐2^2^‐3Myc with MP‐GFP, MP^H67C^‐GFP, MP^N125A^‐GFP, MP^K129Q^‐GFP, MP^A134N^‐GFP, MP^I147M^‐GFP, MP^I168N^‐GFP or GFP were harvested at 24–26 hpi. (c) Co‐IP analysis of Tm‐2^2^ self‐association in the presence of wild‐type or ToBRFV MP mutants. *N. benthamiana* leaves co‐expressing Tm‐2^2^‐3HA and Tm‐2^2^‐3Myc with MP‐GFP, MP^I147M^‐GFP, MP^I168N^‐GFP or GFP were harvested at 36 hpi. Total proteins were immunoprecipitated (IP) with anti‐Myc beads, followed by immunoblotting (IB) with anti‐Myc or anti‐HA antibodies. Input proteins were detected by immunoblotting with anti‐GFP or anti‐HA antibodies. Plants were agroinfiltrated at 3–4 weeks old. For each treatment, three plants were used per treatment, and the experiment was independently repeated three times.

Previous studies showed that Tm‐2^2^ self‐association is essential for HR activation (Wang et al. 2020). We therefore tested whether ToBRFV MP fails to induce Tm‐2^2^ self‐association and whether MP mutants induce it. Tm‐2^2^‐3Myc with Tm‐2^2^‐3HA were transiently co‐expressed with TMV MP‐GFP, ToBRFV MP‐GFP, or empty vector in *N. benthamiana* leaves, followed by Co‐IP at 24–26 hpi (ToBRFV MP‐GFP as a negative control). Results showed that TMV MP‐GFP induced Tm‐2^2^ self‐association, whereas ToBRFV MP‐GFP did not (Figure 6a).

We next examined whether ToBRFV MP‐GFP mutants promoted Tm‐2^2^ self‐association. Transient co‐expression of Tm‐2^2^‐3Myc with Tm‐2^2^‐3HA in the presence of ToBRFV MP‐GFP and its mutants showed that MP^H67C^‐GFP, MP^N125A^‐GFP, MP^K129Q^‐GFP and MP^A134N^‐GFP markedly enhanced Tm‐2^2^ self‐association at 24 hpi, whereas wild‐type ToBRFV MP‐GFP, MP^I147M^‐GFP and MP^I168N^‐GFP had similar weak effects (Figure 6b). As MP^I168N^‐induced weak HR cell death that occurred later than 24 hpi Co‐IP at 36 hpi further revealed that MP^I168N^‐GFP slightly enhanced Tm‐2^2^ self‐association compared to MP‐GFP or MP^I147M^‐GFP (Figure 6c). These results indicate that weak interactions between ToBRFV MP (or MP^I147M^) and Tm‐2^2^ are insufficient to trigger Tm‐2^2^ self‐association, and that residues H67, N125, K129, A134 and I168 are critical for avoiding induction of Tm‐2^2^ self‐association.

### 2.7 H67, N125, K129, A134 and I168 Are Located in a Subdomain of Predicted MP Structure

**FIGURE 7:**
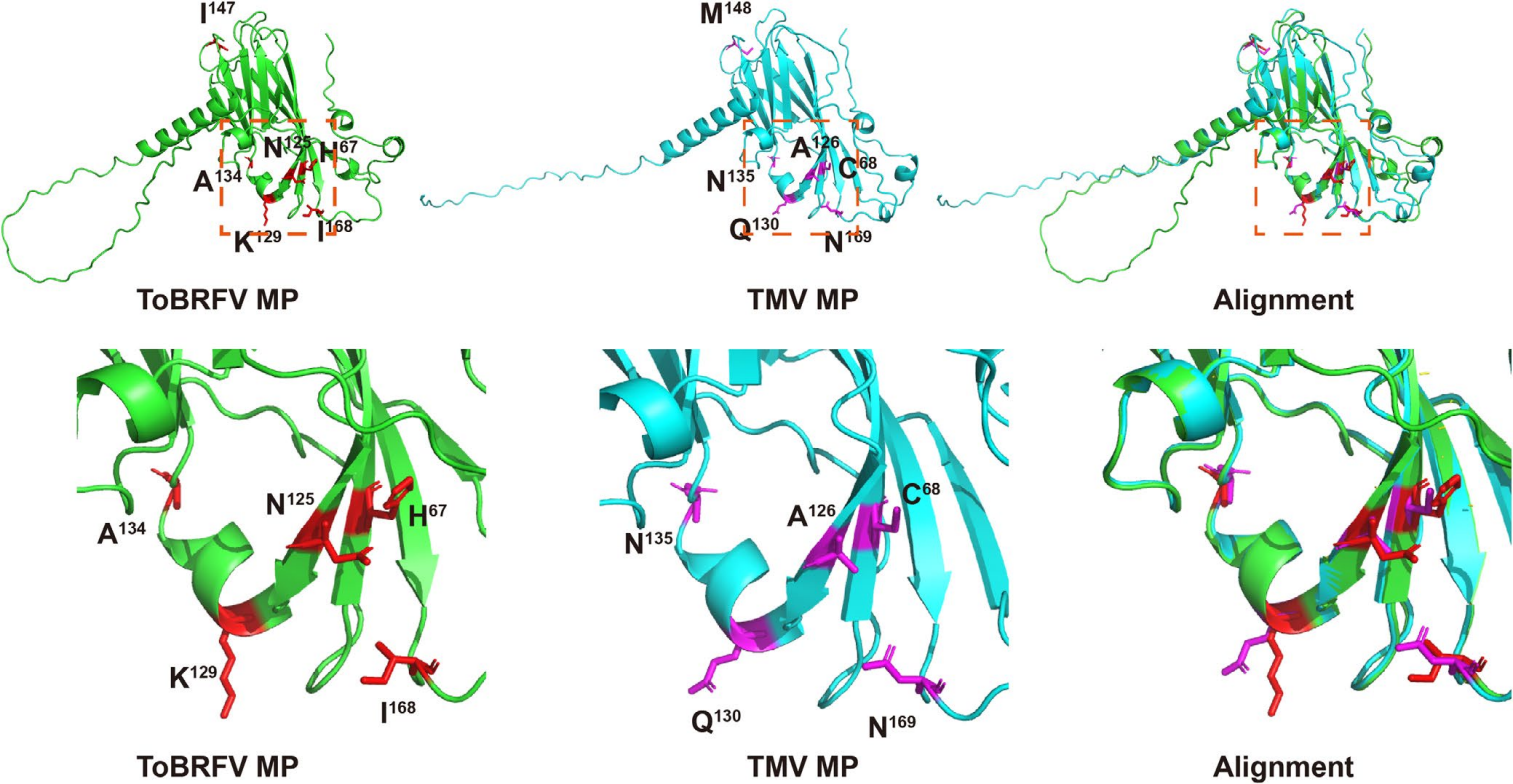
Three‐dimensional structural analysis of the six amino acid residues in tomato brown rugose fruit virus (ToBRFV) MP and the corresponding residues in tobacco mosaic virus (TMV) MP. Three‐dimensional structures were predicted using AlphaFold3, and structural alignment was performed using PyMOL. Residues H67, N125, K129, A134, I147 and I168 in ToBRFV MP are highlighted in red, whereas C68, A126, Q130, N135, M148 and N169 in TMV MP are highlighted in magenta. Close‐up views are shown in the lower panel.

To elucidate the structural basis of ToBRFV MP evasion of Tm‐2^2^‐mediated resistance, predictions of ToBRFV and TMV MP structures were performed using AlphaFold3. Three‐dimensional structure prediction showed that the central regions of ToBRFV MP spanning V34 to I168 and TMV MP spanning C36 to N169 exhibited high pIDDT values (Figure S1). Both ToBRFV MP and TMV MP adopted a typical β‐sandwich fold, with β‐sheets in the central region and α‐helices or loops at both ends. In the ToBRFV MP, residues H67, N125, K129, A134 and I168 were situated within a region comprising segments of three β‐strands, one α‐helix and one loop at one end of the β‐sandwich, whereas I147 was located in the loop at the opposite end (Figure 7). The corresponding residues C68, A126, Q130, N135, M148 and N169 in the TMV MP were located in a similar structural region (Figure 7). Combination of the ToBRFV MP and Tm‐2^2^ interaction results (Figure 5) with the structural model suggests that the subdomain containing H67, N125, K129, A134 and I168 may be critical for evading Tm‐2^2^ recognition.

## 3 Discussion

Plant NLRs recognise viral effectors to activate immune responses and confer resistance, but viruses evade this response by either preventing NLR‐effector interaction or permitting interaction without triggering immune. For example, the NLR Sw‐5b recognises a conserved 21‐amino acid motif in the tospoviral NSm effector to confer broad‐spectrum resistance against most tospoviruses (Zhao et al. 2017). The SD or NB‐ARC domain of Sw‐5b directly interacts with NSm; however, resistance‐breaking mutants such as TSWV NSm^C118Y^ or NSm^T120N^ exhibited reduced affinity for the Sw‐5b SD or NB‐ARC‐LRR domain (Huang et al. 2019). The central region of potato virus Y CP is crucial for the recognition by NLR Ry_sto_. Substitutions S125A, R157D and D201R in the conserved RNA‐binding pocket of potato virus Y CP abolish its interaction with NLR Ry_sto_ and prevent the Ry_sto_‐mediated resistance (Grech‐Baran et al. 2022). In contrast, barley stripe mosaic virus TGB1 interacts with NLR BSR1, but TGB1 mutants R390K and T392K bind BSR1 normally yet fail to activate BSR1‐mediated resistance (Wu et al. 2022). In our study, wild‐type ToBRFV MP showed interactions weakly with Tm‐2^2^ (Figure 5a), while mutations H67C, N125A, K129Q, A134N, I147M and I168N enhanced the MP–Tm‐2^2^ interaction (Figure 5b). Consistent with this, ToBRFV mutants harbouring these changes showed defective or impaired movement in *Tm‐2* ^*2*^‐transgenic *N. benthamiana* plants (Figures 1 and 2), indicating ToBRFV evades Tm‐2^2^‐mediated resistance by attenuating MP–Tm‐2^2^ interaction strength.

Viruses frequently overcome NLR‐mediated resistance via effector mutants that fail to elicit HR. TSWV NSm mutants carrying C118Y, T120N, D122A or R124A substitutions do not trigger Sw‐5b ‐mediated HR, enabling systemic infection of *Sw‐5b*‐transgenic *N. benthamiana* plants (Huang et al. 2022; Zhao et al. 2016). Likewise, ToMV MP mutants harbouring S238R and K244E do not elicit Tm‐2^2^‐mediated HR, allowing systemic infection of tomato plants carrying *Tm‐2* ^*2*^ (Weber et al. 1993; Zhao et al. 2013). Here, we found that MP^H67C^, MP^N125A^, MP^K129Q^, MP^A134N^ and MP^I168N^ strongly induced Tm‐2^2^‐mediated HR and prevented systemic infection by corresponding ToBRFV mutants in *Tm‐2* ^*2*^‐transgenic *N. benthamiana* plants (Figures 1 and 3). Notably, MP^I168N^ elicited only mild HR, correlating with slowed intercellular movement of ToBRFV^I168N^ (Figures 2 and 3). Self‐interaction is essential for Tm‐2^2^‐mediated HR (Wang et al. 2020). The MP^H67C^, MP^N125A^, MP^K129Q^, MP^A134N^ and MP^I168N^ mutants induced Tm‐2^2^ self‐interaction (Figure 6), whereas MP^I147M^ failed to trigger HR, self‐interaction, or restriction of ToBRFV^I147M^ systemic movement at 9 dpi (Figures 1, 3 and 6). These results indicate that ToBRFV evades Tm‐2^2^ by preventing Tm‐2^2^ self‐interaction and HR activation.

Structural modelling positioned H67, N125, K129, A134 and I168 at one end of the β‐sandwich fold, with I147 at the opposite end (Figure 7). Mutations at H67, N125, K129, A134 or I168 triggered Tm‐2^2^‐mediated HR, whereas I147M did not (Figure 3). Tm‐2^2^ mutant Tm‐2^2‐S723Y^ restores recognition of ToBRFV MP and HR induction, but fails against ToBRFV MP^E132K^ (Wang et al. 2025). E132 also resides within the subdomain encompassing H67, N125, K129, A134 and I168 (Figure S2). These findings suggest that this subdomain constitutes a critical interface for Tm‐2^2^ evasion, with I147 potentially acting indirectly to modulate its conformation.

In conclusion, ToBRFV establishes systemic infection in *Tm‐2* ^*2*^‐transgenic *N. benthamiana* by attenuating MP–Tm‐2^2^ interaction strength, thereby preventing Tm‐2^2^ self‐association and activation of Tm‐2^2^‐mediated HR. Our findings imply that engineering Tm‐2^2^ mutants with enhanced affinity for ToBRFV MP could provide effective control of ToBRFV.

## 4 Experimental Procedures

### 4.1 Plant Materials

Transgenic *N. benthamiana* expressing the full‐length *Tm‐2* ^*2*^ gene was kindly provided by Prof. Yule Liu from Tsinghua University (Zhang et al. 2013). Wild‐type and *Tm‐2* ^*2*^‐transgenic *N. benthamiana* plants were grown in a greenhouse at 23°C with a 16‐h light/8‐h dark cycle.

### 4.2 Plasmid Construction

The construction methods of pCBToBRFV and pCBToBRFV‐GFP infectious clones were described before (Ma et al. 2025). For Co‐IP assay, the full‐length fragment of ToBRFV *MP* gene and TMV *MP* gene were amplified and inserted into pCam Pro35S:GFP, while the full‐length fragment of *Tm‐2* ^*2*^ gene was amplified and separately inserted into pCamPro35S:3HA and pCamPro35S:3Myc. Singe‐point mutation vectors were generated using site‐directed mutagenesis as described previously (Liu and Naismith 2008). The primers used in this study are listed in Table S1.

### 4.3 RT‐PCR

Total RNA was extracted from *N. benthamiana* leaves using TRIzol reagent (TransGen Biotech) according to the manufacturer's instructions. First‐strand cDNA was synthesised from the total RNA using a reverse transcription kit (Vazyme). For cloning of viral genes, PCR amplification was performed using Phanta Max Super‐Fidelity DNA Polymerase (Vazyme) with specific primers. The PCR products were analysed via 1% agarose gel electrophoresis.

### 4.4 Agroinfiltration and Transient Expression

*Agrobacterium* cells harbouring different constructs were prepared as previously described (Yan, Cheng, et al. 2021). *Agrobacterium tumefaciens* GV3101 harbouring the indicated binary vectors was cultured in Luria Bertani (LB) medium containing kanamycin and rifampicin at 28°C overnight. The bacteria were harvested by centrifugation at 8000 *g* for 5 min and resuspended in infiltration buffer (10 mM MgCl₂, 10 mM MES, 150 μM acetosyringone). For transient protein expression, the *Agrobacterium* cells were adjusted to OD_600_ = 0.4, while for virus inoculation, the cells were adjusted to OD_600_ = 0.2. The suspensions were incubated at room temperature for 2–3 h prior to infiltration. The bacterial cultures were then infiltrated into the abaxial surface of expanded leaves of *N. benthamiana* plants at 3‐ to 4‐weeks old using a needleless syringe.

### 4.5 Viral Inoculation

For rub‐inoculation, systemic leaves infected with ToBRFV‐GFP and mutants were harvested at 10 dpi. The collected leaves were ground in phosphate‐buffered saline (PBS), using a tissue‐to‐buffer ratio of 1:10 (w/v), and the suspension was gently rubbed with 400‐mesh carborundum onto the expanded leaves.

### 4.6 Western Blotting

Systemic or infiltrated leaf tissues were ground in liquid nitrogen and homogenised in extraction buffer (10% glycerol, 25 mM Tris–HCl, pH 7.5, 1 mM EDTA, 150 mM NaCl, 0.5% v/v NP40, 10 mM dithiothreitol [DTT] and 1 × protease inhibitor cocktail) at a ratio of 1:2 (w/v). Lysates were centrifuged at 12,000 *g* for 5 min, and the supernatants were boiled with 5 × SDS loading buffer for 10 min. Proteins were separated by 10% SDS‐PAGE and transferred to nitrocellulose membranes. The membranes were probed with laboratory‐prepared anti‐ToBRFV CP polyclonal antibodies (1:1000) followed by horseradish peroxidase (HRP)‐conjugated goat anti‐rabbit IgG (1:50,000; Sigma‐Aldrich). Signals were visualised using SuperSignal West Dura substrate (Thermo Fisher Scientific) and a chemiluminescence imaging system (Shenhua Science Technology).

### 4.7 Confocal Microscopy

For cell‐to‐cell movement, GFP was excited at 488 nm and captured in the range of 500–540 nm using a Zeiss LSM800 confocal microscope. The diameter of the infection foci and the relative PD/PM fluorescence intensity of GFP were analysed using ZEN 3.2 blue edition software.

### 4.8 Trypan Blue Staining

The collected infiltrated leaves were boiled for 10 min in staining solution composed of ethanol and trypan blue staining solution (10 mL lactic acid, 10 mL glycerol, 10 mL phenol, 10 mL water and 20 mg trypan blue) at a ratio of 1:2. Subsequently, the leaves were incubated in 2.5 g/mL chloral hydrate destaining solution for approximately 15 h at room temperature.

### 4.9 Co‐IP Assays

Two grams of co‐infiltrated *N. benthamiana* leaves were collected at 24–26 hpi and extracted using extraction buffer (10% glycerol, 25 mM Tris–HCl, pH 7.5, 1 mM EDTA, 150 mM NaCl, 0.5% v/v NP40, 10 mM DTT and 1 × protease inhibitor cocktail). The supernatant was incubated with anti‐GFP agarose beads or anti‐Myc agarose beads (AlpaLifeBio) for 3 h at 4°C and centrifuged at 1900 *g* for 2 min. The beads were then collected and resuspended in 2 × SDS loading buffer and boiled 10 min. The eluted proteins were subjected to immunoblot analysis using anti‐HA (Roche Diagnostics), anti‐GFP (Abways Technology) and anti‐Myc (Abways Technology) antibodies.

### 4.10 Statistical Analyses

Statistical differences (*p* < 0.05) among the treatments were determined using Duncan's multiple range test in SPSS software (v. 19.0). The raw data are provided in Table S2.

### 4.11 Protein Structure Prediction

The three‐dimensional structures of ToBRFV MP and TMV MP were predicted using AlphaFold3 (Google DeepMind). The full‐length amino acid sequences of the respective MPs were used as input. Structural visualisations and comparisons were performed using PyMOL (Schrödinger LLC).

## Author Contributions

**Hua‐Yu Ma:** conceptualization, methodology, investigation, writing – original draft, data curation. **Yi‐Jie Liu:** investigation, data curation. **Xiao‐Xue Huan:** investigation, data curation. **Shu‐Yu Liu:** investigation, data curation. **Xiu‐Qi Mu:** investigation, data curation. **Li‐Meng Zhang:** investigation, data curation. **Zhi‐Long Bao:** data curation. **Yan‐Ping Tian:** methodology. **Zhi‐Yong Yan:** conceptualization, methodology, data curation, supervision, funding acquisition, writing – original draft, writing – review and editing. **Xiang‐Dong Li:** conceptualization, writing – review and editing, supervision, funding acquisition, methodology.

## Funding

This study was supported by grants from the Natural Science Foundation of Shandong Province (ZR2023QC135), National Natural Science Foundation of China (32302322, 32472523), China Postdoctoral Science Foundation (2024M751874), Key R&D Program of Shandong Province, China (2023LZGC013), Taishan Scholar Construction project (No. ts2022028), Agricultural scientific and technological innovation project of Shandong Academy of Agricultural Sciences (CXGC2025A01).

## Conflicts of Interest

The authors declare no conflicts of interest.

## Supporting information


**Figure S1:** Structural models of ToBRFV MP and TMV MP. The structures were predicted using AlphaFold3. The predicted pLDDT scores range from 0 to 100, with pLDDT > 90 indicating very high confidence, 90 > pLDDT > 70 indicating confidence, and 70 > pLDDT > 50 indicating low confidence. Scores below 50 suggest extremely low confidence, indicating that accurate prediction may not be possible. An expected position error (EPE) plot showing residue alignment in the predicted structure. The colour gradient represents position error in Ångströms, with darker green indicating better alignment and lower error.

**Figure S2:** E132 is located within the subdomain that contains residues H67, N125, K129, A134 and I168 in ToBRFV MP. Residues H67, N125, K129, A134 and I168 are highlighted in red, whereas E132 is highlighted in yellow.

**Table S1:** Primers used in this study.

**Table S2:** Statistical analyses in this study.

## Data Availability

Data sharing not applicable to this article as no datasets were generated or analysed during the current study.
