# Supplementary figures and images for: Tomato Brown Rugose Fruit Virus Evades Tm‐22‐Mediated Resistance by Avoiding the Induction of Tm‐22 Self‐Association
Source: Mol Plant Pathol. 2026 Mar 19;27(3):e70243. doi: 10.1111/mpp.70243 (PMC13098148; doi:10.1111/mpp.70243)

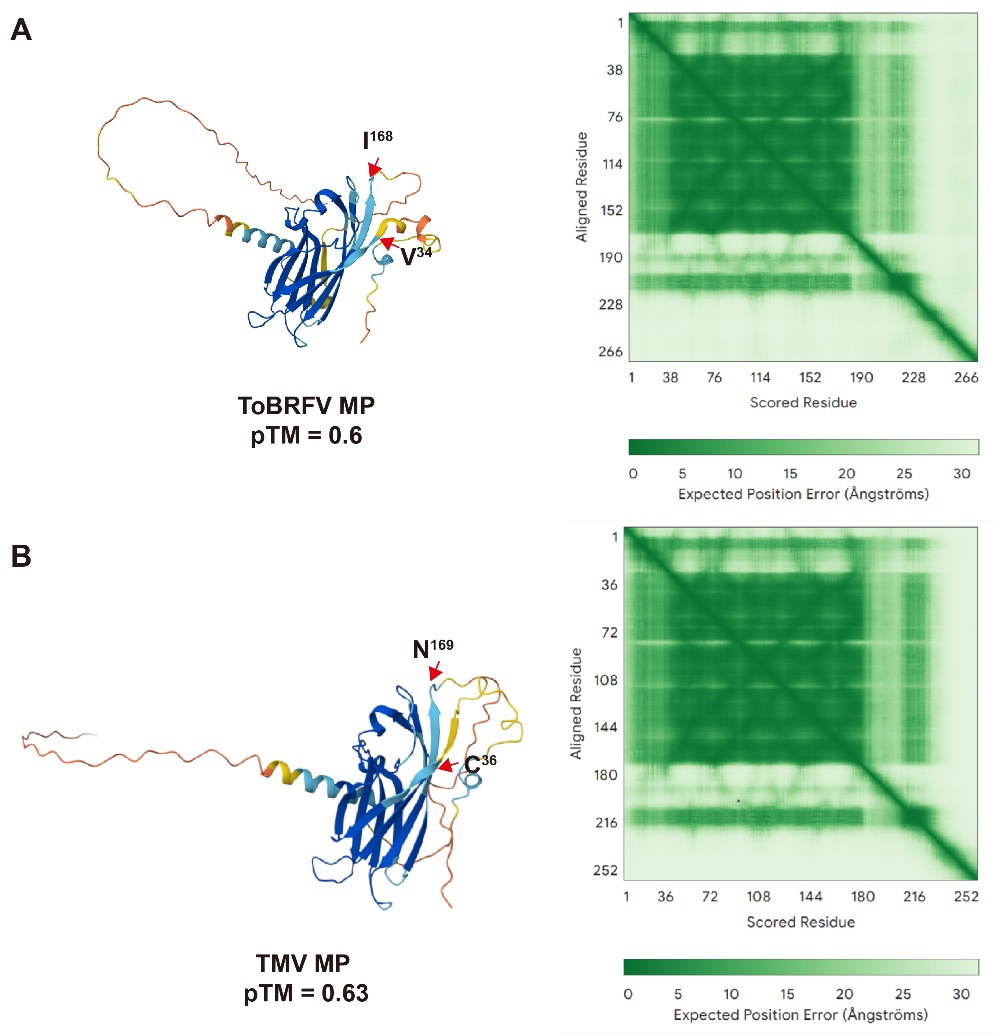


Figure S1

Supplement: Supplementary file 1 — Figure S1: Structural models of ToBRFV MP and TMV MP. The structures were predicted using AlphaFold3. The predicted pLDDT scores range from 0 to 100, with pLDDT > 90 indicating very high confidence, 90 > pLDDT > 70 indicating confidence, and 70 > pLDDT > 50 indicating low confidence. Scores below 50 suggest extremely low confidence, indicating that accurate prediction may not be possible. An expected position error (EPE) plot showing residue alignment in the predicted structure. The colour gradient represents position error in Ångströms, with darker green indicating better alignment and lower error. [file MPP-27-e70243-s003.docx]

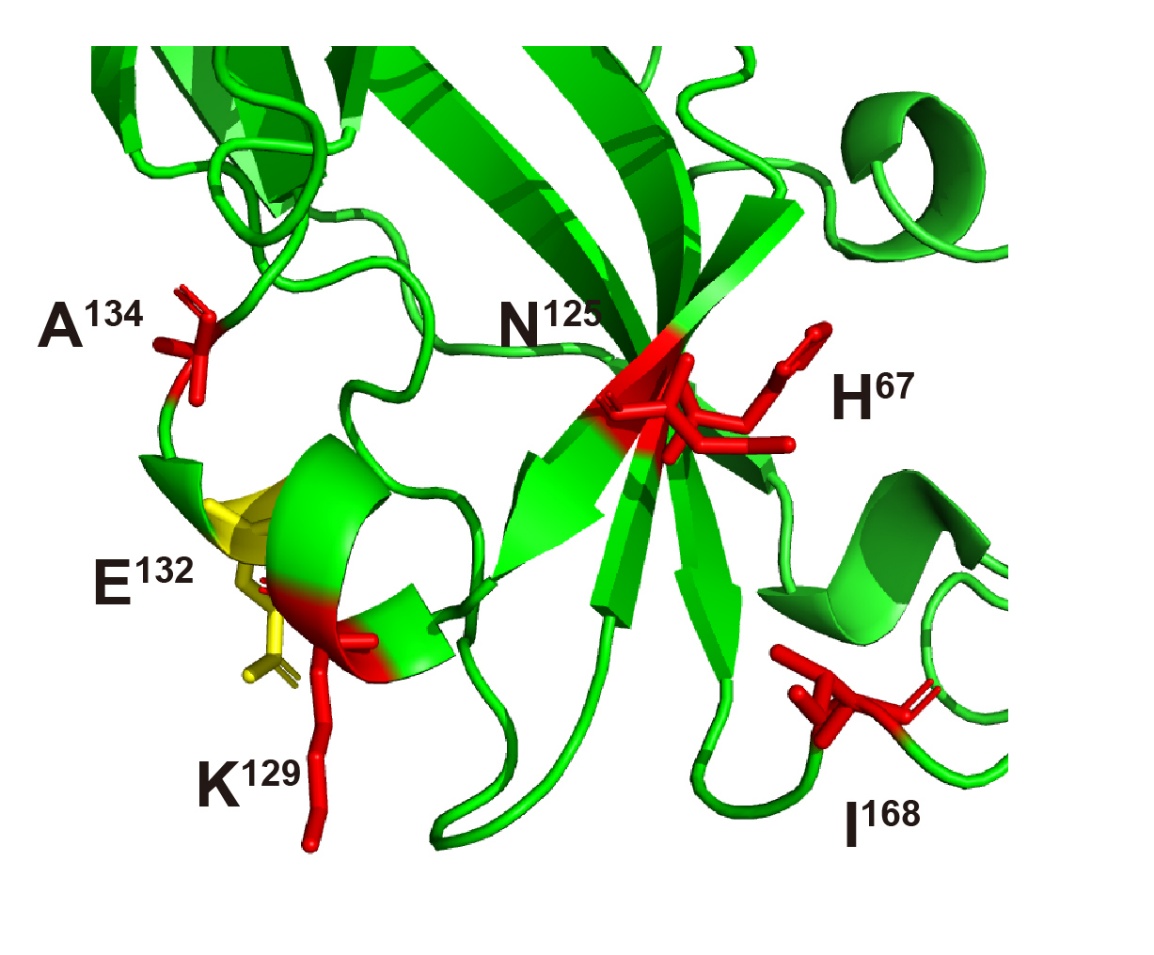


Figure S2

Supplement: Supplementary file 2 — Figure S2: E132 is located within the subdomain that contains residues H67, N125, K129, A134 and I168 in ToBRFV MP. Residues H67, N125, K129, A134 and I168 are highlighted in red, whereas E132 is highlighted in yellow. [file MPP-27-e70243-s001.docx]
